# Bacterial sex in dental plaque

**DOI:** 10.3402/jom.v5i0.20736

**Published:** 2013-06-03

**Authors:** Ingar Olsen, Gena D. Tribble, Nils-Erik Fiehn, Bing-Yan Wang

**Affiliations:** 1 Faculty of Dentistry, Department of Oral Biology, University of Oslo, Oslo, Norway; 2 Department of Periodontics, School of Dentistry, University of Texas Health Science Center at Houston, Houston, TX, USA; 3 Faculty of Health Sciences, Department of International Health, Immunology and Microbiology, University of Copenhagen, Copenhagen, Denmark

**Keywords:** bacteria, bacterial sex, DNA transfer, dental plaque

## Abstract

Genes are transferred between bacteria in dental plaque by transduction, conjugation, and transformation. Membrane vesicles can also provide a mechanism for horizontal gene transfer. DNA transfer is considered bacterial sex, but the transfer is not parallel to processes that we associate with sex in higher organisms. Several examples of bacterial gene transfer in the oral cavity are given in this review. How frequently this occurs in dental plaque is not clear, but evidence suggests that it affects a number of the major genera present. It has been estimated that new sequences in genomes established through horizontal gene transfer can constitute up to 30% of bacterial genomes. Gene transfer can be both inter- and intrageneric, and it can also affect transient organisms. The transferred DNA can be integrated or recombined in the recipient's chromosome or remain as an extrachromosomal inheritable element. This can make dental plaque a reservoir for antimicrobial resistance genes. The ability to transfer DNA is important for bacteria, making them better adapted to the harsh environment of the human mouth, and promoting their survival, virulence, and pathogenicity.

In an excellent review on the origins and evolution of antibiotic resistance, Davies and Davies (1) recently proposed that the intestinal tract of animals and humans is a bordello for microorganisms since bacterial transfer of resistance genes occurs here *ad libitum*. Indeed, recent studies have found diverse antibiotic resistance genes in the human gut microbiome, indicating such transfer (2). Bacterial conjugation has long been regarded as the equivalent of sexual reproduction or mating in bacteria since an exchange of genetic material is involved. Also, sex pheromones that induce mating responses in bacteria have been detected (e.g. in commensal oral streptococci) (3). They may promote intergeneric gene acquisition among bacteria in oral biofilms. The close proximity of cells in the dense and constricted bacterial populations of multispecies dental biofilm facilitates plasmid dispersal through conjugation (4, 5). In biofilm-grown *Streptococcus mutans*, cells were transformed at rates 10- to 600-fold higher than in planktonic *S. mutans* cells (6). Furthermore, DNA release and transformation seem to be parts of the biofilm-related life cycle, and released DNA has the capacity to stabilize biofilm structure and architecture (7). The source for this genetic material is probably extracellular DNA (eDNA) derived from biofilm cells or transitory bacterial cells reaching the mouth (7). Clearly, genes are transferred between bacterial cells in dental plaque with enhanced efficiency, but does this phenomenon imply that dental plaque can be considered a bordello for plaque bacteria? This review focuses on bacterial sex in dental plaque.

## Bacterial sex

Bacteria are predominantly asexual and reproduce by binary fission. A single cell hereby replicates its genome and divides into two identical daughter cells. Variations in this process usually occur by mutation or duplication of existing intragenomic information (8). However, the genetic information of bacteria can be expanded and modified through mechanisms that introduce DNA from external sources. Bacterial sex is defined as the inheritance of DNA from any source aside from a bacterium's one parent cell (8).

Most bacterial species consist of a huge population of strains, which genetically are not very similar. Therefore, a bacterial species can be described by its pan-genome, which includes the core genome containing the genes that are present in all strains of the species, a dispensable genome containing genes that are present in two or more strains, and genes that are unique to single strains (9). Bacterial sex can take place between strains to recombine dispensable and unique genes into a new ‘offspring.’ In contrast to sex among eukaryotes, sex in bacteria is unidirectional and does not involve gamete fusion or reproduction. The acquirement of DNA through bacterial sex causes increased diversity through the alteration of existing genes and the introduction of new DNA sequences. *Streptococcus* is an example of a genus that is very diverse, with high levels of gene gain and loss (i.e. with extreme levels of evolutionary plasticity) (10). This genetic diversity is the result of mainly horizontal gene transfer in biofilms occurring as conjugation, transduction, and transformation (discussed later in this review).

The formation of new genotypes by homologous recombination is commonly referred to as ‘bacterial sex’ because the result is similar to that of higher eukaryotes: production of offspring with a mixture of traits from each parent. Bacterial sex can also introduce a limited amount of new sequences to bacteria through horizontal gene transfer of mobile elements, and increasing the gene repertoire of the recipient may change the virulence of the bacteria (e.g. through establishment of pathogenicity islands, new metabolic pathways, and plasmids or transposons encoding antibiotic resistance). Actually, sex and virulence are intimately integrated in a wide variety of microbes (11). The majority of genes in bacterial genomes seem to have been acquired by horizontal gene transfer at some time during the evolutionary history of the lineage, whereas the diversity of gene repertoires in eukaryotes is mainly due to gene duplication and loss (12).

## DNA transfer between bacterial cells

DNA can be transferred between bacterial cells mainly by transduction, conjugation, or transformation. Transduction is caused by bacterial viruses, conjugation by a bacterial sex pilus, and transformation through DNA uptake by naturally competent bacteria (13). After transfer, DNA can be physically recombined into the chromosome by various cytoplasmic proteins or, in the case of a plasmid, maintained as a separate molecule. A number of the bacterial genomes that have been sequenced contain genes acquired through horizontal gene transfer.

In *transduction*, bacteria-infecting viruses (bacteriophages) introduce their own or sometimes alien DNA into the new host's genome. Transduction requires specific receptors on the cell surface of the recipient bacterium. The amount of DNA that can be packed within a phage can be about 100 kb but varies greatly. The proteins of the bacteriophage protect its double-stranded DNA from destruction by host endonucleases. Bacteriophages can become dormant after integration in the host chromosome (temperate), or they can cause lysis of the host (lytic). Bacterial viruses, together with eukaryote-infecting viruses, may be the most common sources of genetic material on the planet (8).

*Conjugation* requires cell-to-cell contact. In conjugation, DNA is transferred between two metabolically active cells by self-transmissible and mobilizable plasmids called F (sex factor) plasmids, integrative conjugation elements (plasmids integrating into a chromosome producing high-frequency recombinant (Hfr) strains), or conjugative transposons (which encode proteins for their excision and transposition into recipient strains) (8). Conjugation can result in the exchange of homologous or heterologous stretches of DNA between the mating pair. Conjugative transposons occur frequently in Gram-positive bacteria but have also made their way to Gram-negative organisms because they are highly evolved for transfer among a broad host range. Their omnipresence makes them important players in the dissemination of a large variety of antibiotic resistance determinants (14). The high density of cells in biofilms increases the spread of plasmids by conjugation, and conjugation itself may stimulate biofilm formation (15).

*Transformation* is the process driven by cells that display competence, which is the ability to take up DNA from the environment. The development of competence involves a quorum-sensing process encoded by different *com* genes. Cells that are competent are able to bind free double-stranded DNA from the environment to cell surfaces, transfer one strand of the DNA across the cell membrane, and then integrate the DNA into the chromosome through recombination with its homologous counterpart. Many bacterial genera in the mouth are naturally competent at all times (e.g. *Haemophilus*, *Campylobacter*, and *Neisseria*), while others such as *Streptococcus* are competent only during specific physiological states (16). A likely source of free DNA in the dental plaque is the matrix, or slime layer, that surrounds bacteria in a biofilm. DNA released by dead lyzed bacteria can be found in the matrix, as well as DNA secreted by living members of the community, to provide structural support for the slime. Transformation by eDNA has been shown to mediate transfer of antibiotic resistance in an oral biofilm model (16).

### Gene transfer among Gram-negative bacteria via membrane vesicles

**Fig. 1:**
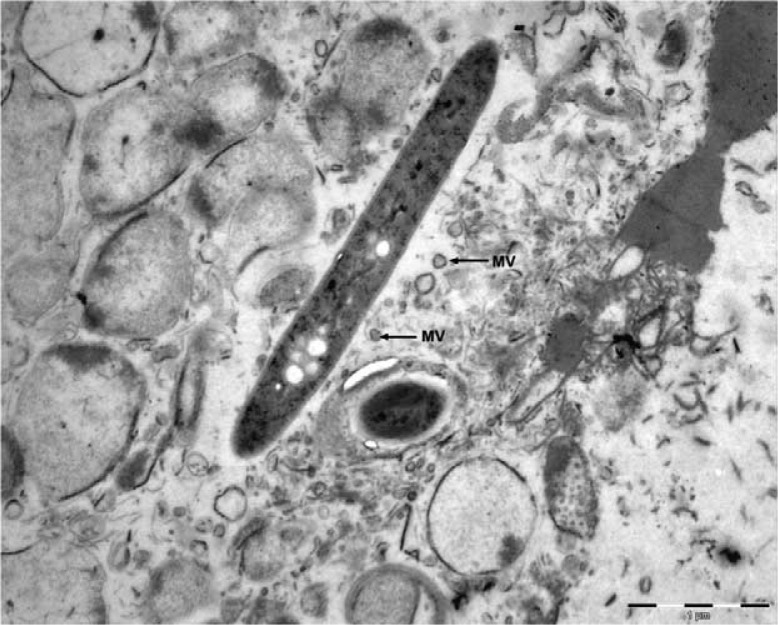
Transmission electron micrograph of a *Leptotrichia buccalis* cell with derived membrane vesicles (MVs) that provide an alternative mode of gene transfer. Bar=1 µm. Courtesy of Emenike R.K. Eribe.

Many Gram-negative bacteria, including bacteria in dental plaque, form and release membrane vesicles (MVs) (Fig. 1) during growth (17–20). Using *Pseudomonas aeruginosa* as a model system, Beveridge et al. (21) demonstrated that Gram-negative bacteria can segregate and package periplasmic components into MVs. These MVs can protect the genetic content that is packaged inside from harsh environmental conditions such as DNase digestion (20, 22). MVs from Gram-negative bacteria can fuse into the surfaces of other species, indicating the possibility of MVs as delivery vehicles for transferring genetic materials and virulence factors from donor to recipient (10, 18). Indeed, it has been reported that MVs transfer virulence genes to recipient Gram-negative bacteria of the same or different species (17, 19, 20, 22). Because of their small dimensions (approximately 20–100 nm) (20), MVs could easily reach inaccessible areas such as the interior of biofilms and transport protected DNA to other bacteria, even when the donors and recipients are not in direct contact. These studies suggest that MV release by Gram-negative bacteria represents a novel mechanism for the transfer of genetic materials by these bacteria.

## Dental plaque and microbiome DNA exchange

**Fig. 2:**
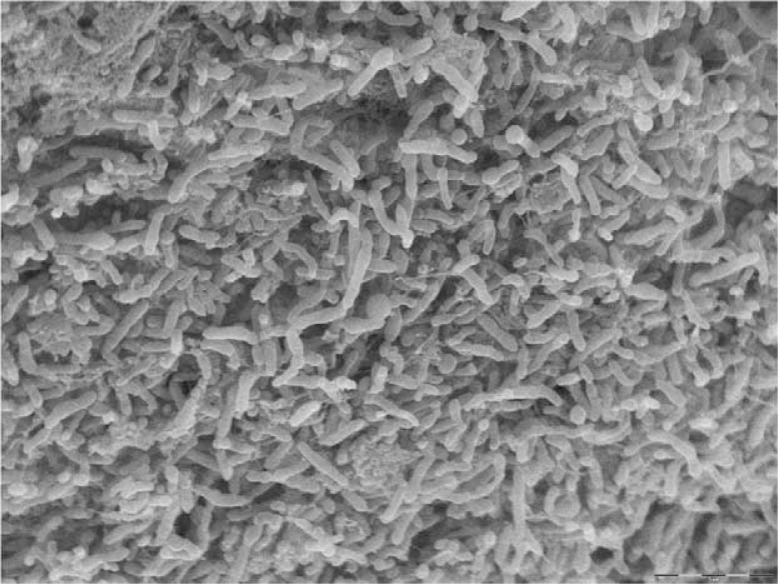
Scanning electron micrograph of a multispecies subgingival biofilm *in vivo* from adult periodontitis. Bar=5 µm. Courtesy of Steinar Stølen.

Dental plaque is a biofilm consisting of multiple bacterial species (Fig. 2). Since the tooth surface is nonshedding, this biofilm is actually the most diverse one in the human body in terms of microorganisms. With pyrosequencing, which detects even rare organisms at low concentrations, 1000 species have been found in plaque (23). This is in contrast to the previous estimate of more than 500 species in the dental biofilm (24). With these large numbers of bacteria in constant contact with each other, and with other bacteria transiently passing through the oral cavity, the potential for gene transfer is high. Recent metagenomic and bioinformatic studies have confirmed that horizontal DNA transfer is a common occurrence in human microbiota, and bacteria of the oral cavity are major players in this process (25, 26). In fact, horizontal DNA transfer was detected as commonly occurring between members of the oral microbiota and other bacteria in the body, especially those in the intestine such as the *Bacteroides*. As the oral cavity is the entry point for the entire digestive tract, oral bacteria will be in contact not only with other oral bacteria but also with intestinal bacteria that are transiently passing through the mouth and environmental bacteria that are present in food and drink products. As such, the oral microbiota could represent a reservoir for the exchange of virulence genes, such as genes for antibiotic resistance.

For example, DNA from a transient *Phytoplasma* species, which is normally associated with plants, has been detected in saliva (27). DNA is able to survive in a neutral pH environment, including saliva, for various time lengths (28, 29). Accordingly, the transfer of environmentally derived antibiotic resistance genes into oral bacteria could take place by transformation in addition to conjugation, and the subsequent spread of the newly acquired antibiotic resistance genes could then occur within the plaque population. Also, genetic material could then be transferred into intestinal genera that are only transient in the mouth. In this way, the normal microbiota in the mouth can potentially mediate the transfer of resistance genes between diverse sources and recipients (30). This is similar with findings from the colon, where extensive gene transfer has been found to occur within the genus *Bacteroides* and among *Bacteroides* species and Gram-positive bacteria (31). The colon is another body site that is considered highly conductive to horizontal gene transfer and to stable maintenance of transferred resistance.

## DNA transfer in major dental plaque bacteria

By determining genome sequences of *Porphyromonas gingivalis*, a major pathogen in adult periodontitis, Naito et al. (32) detected extensive genomic rearrangement between the strains ATCC 33277^T^ and W83. Both strains contained large numbers of mobile elements like insertion sequences, miniature inverted-repeat transposable elements, and conjugative transposons frequently connected with genomic rearrangements. Plasmid or chromosomal DNA can be transferred within and between strains of *P. gingivalis* (33). Strains W83 and ATCC 33277 can exchange chromosomally located antibiotic resistance markers. Natural competence was the major driving force behind DNA exchange in *P. gingivalis*, while conjugative DNA transfer played a minor role (33). Although DNA transfer in *P. gingivalis* is complex, *comF-*dependent transformation by uptake of eDNA appears to be the most important mechanism of DNA transfer in strain W83. Additionally, Tribble et al. (34) demonstrated the presence of eDNA in *P. gingivalis* biofilms. eDNA is predicted to be the source of the DNA exchanged between different strains during cohabitation in biofilms. *comF* is critical for DNA uptake from the environment. A transcriptome comparison analysis of planktonic and biofilm-cultured *P. gingivalis* showed that in the latter, *comF* expression increased twofold (35). Also, studies with other bacteria have indicated that eDNA is an important part of biofilm matrixes (36–39). The DNA exchange between strains may be important for the evolution of *P. gingivalis* from a commensal to a pathogenic state. It is also likely to be important for the short-term survival of *P. gingivalis* in the periodontal pocket, a stressful and highly competitive habitat. Changes in antigens obtained through genetic transfer may facilitate survival of the organism through protection against the adaptive immune responses.

**Fig. 3:**
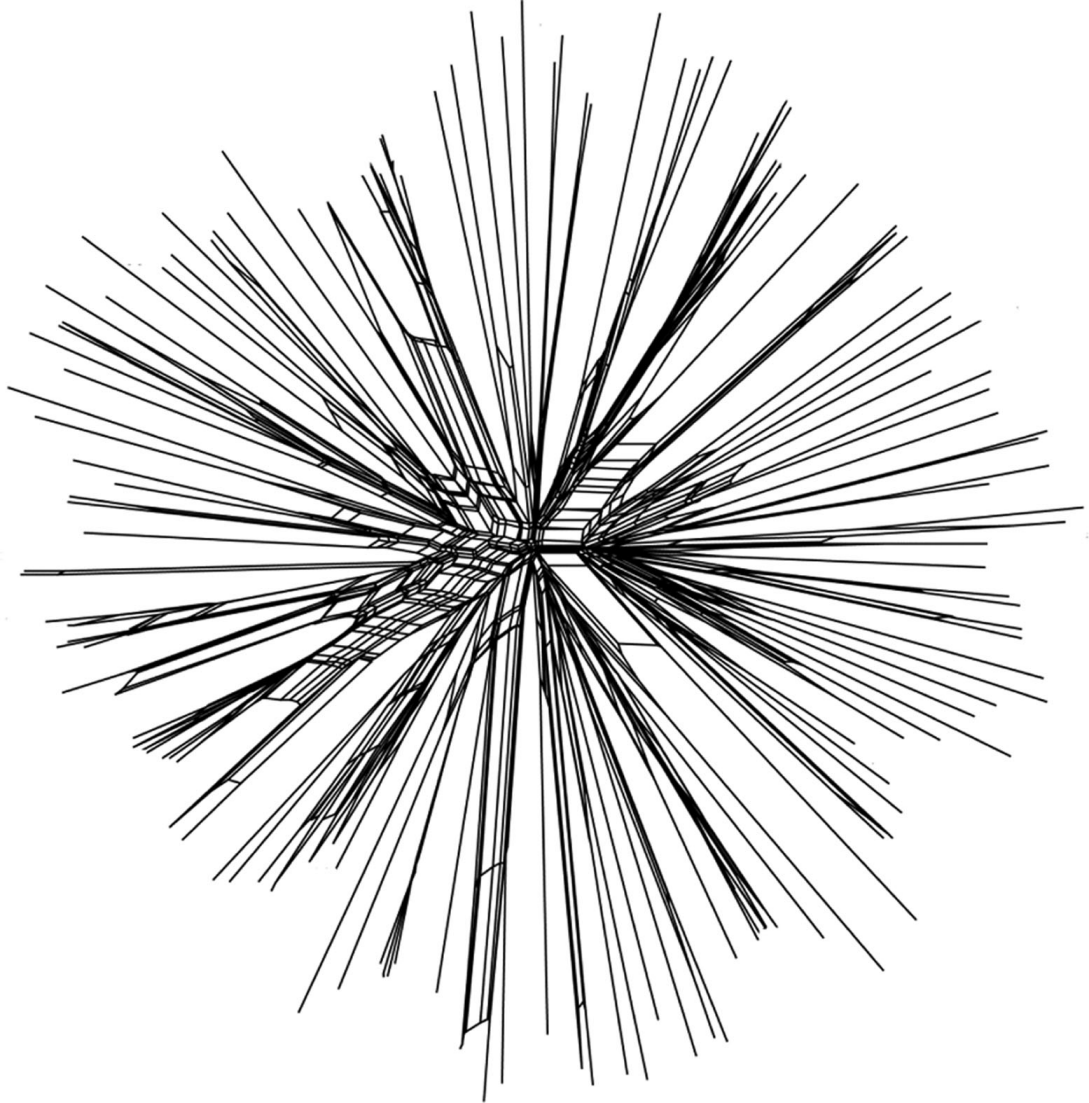
Split decomposition analysis graph constructed by Split Tree (v. 4.6) (www.splitstree.org) from allelic profiles of the 138 sequence types (STs) of *P. gingivalis* in the multilocus sequence typing (MLST) database. Recombination is illustrated by the net- or bushlike structures in the center. The star- or treelike structures indicate clonality. From Reference [40]. Courtesy of Morten Enersen.

**Fig. 4:**
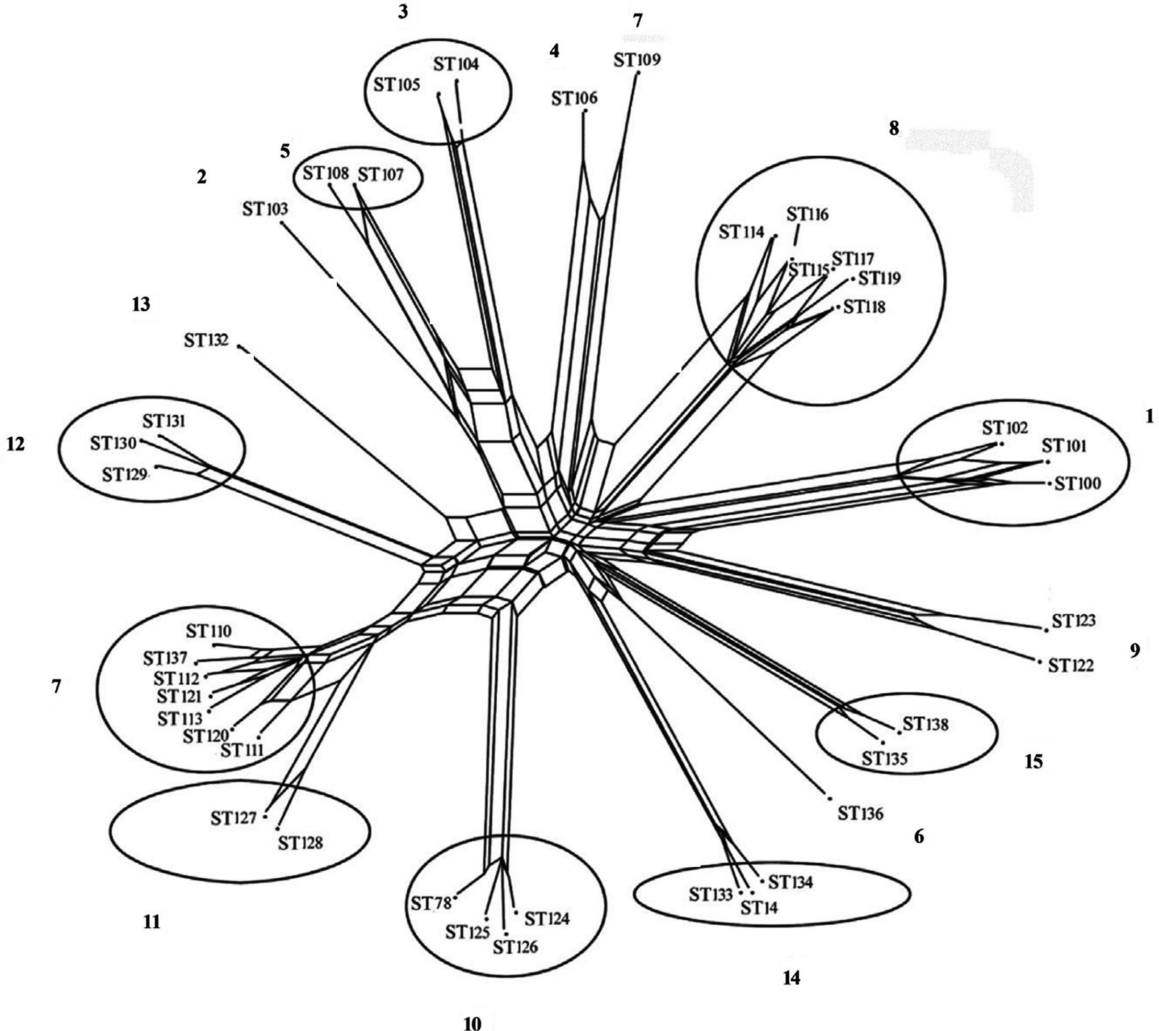
The degree of recombination between 93 isolates of *P. gingivalis* sampled *in vivo* from 15 single sites of ‘refractory’ periodontitis patients is illustrated by a SplitsTree graph with superimposed results of the eBurst analysis to the graph. Patient and site numbers are marked in bold. Several STs detected in each periodontal site are located in groups (circles and ellipses) except for patients 2, 4, 6, 7, 9, and 13, for whom only one ST was found per site. See text in Reference [40]. Courtesy of Morten Enersen.

A multilocus sequence typing (MLST) scheme for *P. gingivalis* has been established (www.pubmlst.org/pgingivalis). It indicates a high degree of genetic diversity and a weakly clonal population structure comparable to that seen in *Neisseria meningitidis* (40) (Fig. 3). Multiple sequence types (STs) were detected in one site in several patients with ‘refractory’ periodontitis (Fig. 4). This reflected allelic variation in two housekeeping genes and recombination between different clones *in vivo* in subgingival plaque of the periodontal pocket (40). Accordingly, the genetic variation in *P. gingivalis* strains is considerable and reinforces the concept that exchange of genes occurs in subgingival plaque to improve the chances of *P. gingivalis*’ survival.

Plasmids may play an important role in horizontal gene transfer in fusobacteria. Genetic analyses of *Fusobacterium nucleatum*, which is another opportunistic pathogen implicated in periodontitis, demonstrate considerable diversity. Horizontal gene transfer is thought to play a prominent role in the evolution of this species. Several plasmids of *F. nucleatum*, including pFN1, have relaxase gene homologs that may operate in plasmid mobilization. Recently, Claypool et al. (41) found that the fusobacterial plasmid pFN1 encodes genes, enabling efficient mobilization between strains of *Escherichia coli* by the broad-range plasmid RP4. A role was suggested for plasmid mobilization in DNA transfer in *F. nucleatum*. Plasmid or plasmid-related sequences were identified in as many as 11.5% of the strains in a panel of clinical *F. nucleatum* isolates (41). Roberts and Lansciardi (42) showed that two strains of *F. nucleatum* were able to transfer tetracycline resistance by conjugation encoded by the *tet*(M) determinant to strains of *F. nucleatum*, *Peptostreptococcus anaerobius*, and *Enterococcus faecalis*. Another study showed that *P. anaerobius* could transfer *tet*(M) to recipients of *P. anaerobius* and *F nucleatum* (43).

*Veillonella dispar* was shown to transfer the conjugative transposon Tn*916* to four different *Streptococcus* species in a multispecies oral biofilm established in a constant-depth biofilm fermenter (16). In the same system, purified *V. dispar* DNA conferred tetracycline resistance to *S. mitis*. None of the consortia members could develop tetracycline resistance when they were grown as monocultures. In a model oral biofilm established in another constant-depth film fermenter, the conjugative transposon Tn*5397* was transferred from *Bacillus subtilis* to a *Streptococcus* species (44).

Transfer of conjugal elements in oral black-pigmented *Prevotella* species (*P. denticola* and *P. intermedia*) conferred tetracycline and penicillin resistance in the absence of plasmids (45). Transverse alternating-field electrophoresis of restricted chromosomal DNA from the transconjugants revealed rearrangements that indicated that the transfer occurred at strongly preferred sites in the recipient chromosome's genes. In another *Prevotella* study, a small conjugative transposon was identified that encoded a *tet*(Q) gene identical to genes found in the intestinal *Bacteroides* (46). Although the *tet*(Q) gene was the same, the *Prevotella* mobile element was an IS21 transposon not found in the *Bacteroides*, indicating that the *Prevotella* reassembled the resistance gene ‘module’ into its own transposon.

Transfer of a nonconjugative plasmid from *Treponema denticola* to *Streptococcus gordonii* was demonstrated in mixed-species biofilms (47). Transformation of *S. gordonii* occurred using the erythromycin resistance (Ermr) shuttle plasmid pKMR4PE as a donor source. Possibly there is a naturally occurring genetic transfer system within oral spirochetes, or they have the ability to take up and maintain mobilizable plasmids (48). Upregulation of various putative pro-phage genes and transposases was seen in a continuous-culture biofilm model of *T. denticola* ATCC 35405^T^ (49). Also, a functional bacteriophage was isolated from this organism.

Transfer of genetic information in *Actinomyces* species was achieved by transfection with the genomic DNA of bacteriophages from human dental plaque (50). A study concerning a genetic transfer system for *Actinomyces* spp. has shown that multiple genes are involved in fimbriae synthesis and function (51). Furthermore, genetic analysis found various enzymes of strains of *Actinomyces* involved in the adherence of fimbrial receptors on host cells. These circumstances may determine the selection of *Actinomyces* spp. in certain ecological niches in the oral cavity.

In *Aggregatibacter actinomycetemcomitans* strains, Willi et al. (52) observed transduction of antibiotic resistance markers by temperate bacteriophages. Periodontal isolates of *A. actinomycetemcomitans* have been found to contain bacteriophages (53–55). The *tet*(B) determinant was transferable *in vitro* between isolates of *A. actinomycetemcomitans* and a *Haemophilus influenza* recipient and was probably associated with conjugative plasmids (56). Natural competence in some *A*. *actinomycetemcomitans* strains also takes place. *UrpA* is a novel gene that is required for natural competence in *A. actinomycetemcomitans* and is different from corresponding natural-competence genes in other bacterial species (57). A recent study demonstrated differences in full genome content and gene expression between *A. actinomycetemcomitans* strains of the JP2 and non-JP2 genotypes (58). This information might help us understand the pathogenic mechanisms of this important periodontopathogen. Also specific geographic distribution has been observed for the JP2 strain, suggesting limited genetic and species transfer between individuals from different subpopulations (59).

Several streptococcal species enter a physiological state of competence that allows them to bind, transport, and incorporate DNA into their chromosomes. Thereby, foreign DNA from the environment is acquired without cell–cell contact. Competence is linked to biofilm formation through generation of the quorum-sensing molecule competence-stimulating peptide (CSP) in *S. pneumoniae*, *S. mutans*, and *S. gordonii*. Biofilm communities may show peaks in competence over defined time periods or growth rates (60). The acquisition of exogenous DNA by naturally competent oral streptococci might enhance their survival under environmental stress. In *S. gordonii*, a genetic determinant encoded a peptide with activity similar to that of the sex pheromone cAM373 in *E. faecalis*, which promoted intergeneric DNA transfer (3). Interspecies genetic exchange in streptococci has also been demonstrated through the appearance of so-called mosaic genes, which are partially derived from donor DNA and the host chromosome. Additionally, it is clear from the genome of *Streptococcus sanguinis* that a 70 kb region-encoding pathway for vitamin B_12_, biosynthesis, and degradation of ethanolamine and propanediol has been acquired through horizontal gene transfer (61).

Roberts et al. (62) showed in microcosm dental plaque that conjugative transposon Tn*916*-like elements in four tetracycline-resistant streptococcal species could be transferred to other streptococci in filter-mating experiments and within biofilms in a fermenter. This was the first demonstration that a native conjugative transposon could be transferred within a model oral biofilm and that oral streptococci harbor and disseminate these mobile elements to other components of the oral microbiota. Particularly, *S. pneumoniae* and related streptococci are capable of exchanging genes. While oral streptococci can act as donors of DNA for pneumococci, the opposite can also occur, emphasizing the fact that streptococci are genetic reservoirs for transient organisms on their way through the mouth.

A transferable tetracycline resistance Tn*916*S element was characterized in *S. intermedius* (63), the genetic support of which was a conjugative transposon closely related to Tn*916*. Therefore, Tn*916* is apparently involved in the dissemination not only of *tet*(M) but also of *tet*(S). Also, erythromycin resistance genes are prevalent in oral streptococci. Such isolates can be resistant to erythromycin and tetracycline simultaneously (64).

Warburton et al. (65) reported the transfer of antimicrobial resistance from one oral streptococcus species to another during systemic antibiotic treatment of periodontitis. In this case, an *S. cristatus* strain acquired a novel conjugative transposon CTn*6002* that was derived in part from Tn*916* during treatment, making it resistant to doxycycline and erythromycin.

*S. mutans*, which is hypertransformable when grown in biofilms, uses a peptide pheromone quorum-sensing signal transduction system for promoting the uptake and incorporation of foreign DNA (6). Seven *ComC* alleles encoding for three CSPs have been observed in *S. mutans* (66). Kuramitsu and Trapa (67) demonstrated that this organism could be a donor of plasmid DNA that gave rifampicin and erythromycin resistance to *S. sanguinis* and *S. milleri*. Also, transfer of genetic information from *S. sanguinis* to *S. milleri* was seen. Furthermore, fluoride resistance could be transferred between *S. mutans* strains (68) and chromosomally encoded genes providing resistance to rifampin, streptomycin, and spectinomycin as well as plasmids conferring resistance to tetracycline and erythromycin (69). The ability of streptococci to mediate interspecies DNA transfer seems to be common, as a filter-mating study showed that 4 of 12 isolates of oral streptococci were able to transfer genes encoding erythromycin resistance [*erm*(B)] and tetracycline resistance [*tet*(M)] to *E. faecalis* (70).

Mutant genotypes can also accumulate within dental biofilms. In *S. mutans*, natural selection but not increased mutation rate was found to be the primary driving force behind the accumulation of deletion mutants (71). The JP2 clone strains of *A. actinomycetemcomitans* constitute a unique clonal type that includes a 530 base pair deletion in the leukotoxin operon that is implicated in the enhanced leukotoxic activity of the clone (72).

## Interference of gene transfer in dental plaque

Oral bacteria compete with each other for limited nutrients in the oral cavity. Therefore, it is not surprising that some products from different species can interfere with the gene transfer process of another species, since homologous recombination of foreign DNA into the host chromosome may give the recipient survival advantages. It has been demonstrated that proteases produced by both early and late colonizers of dental plaque can inactivate the *S. mutans* CSP, an essential factor to initiate competence in *S. mutans*’ natural transformation, which renders this naturally transformable species incompetent in horizontal genetic transfer (73, 74).

Another example of DNA transfer resistance and interference is found in *Prevotella*. Introduction of foreign DNA into *Prevotella* species is notoriously difficult, and some ruminal strains of *Prevotella* have been shown to produce extracellular endonucleases and high levels of cytoplasmic restriction and modification proteins (75, 76). The presence of secreted DNA nucleases could be predicted to interfere with DNA transfer within the local biofilm environment, via the degradation of eDNA. This could impact not only the strain secreting the DNase but also immediate neighbors that are competent and ‘in the market’ for eDNA. Many oral bacterial strains aside from the *Prevotella* ones have been found to secrete extracellular DNases (77).

## Concluding remarks

There is no doubt that genes are transferred between bacteria in dental plaque, probably by all three major mechanisms of horizontal gene transfer: transduction, conjugation, and transformation. Several examples have been given in this review, which is not meant to be exhaustive. Of particular interest is that dental plaque can be a reservoir for antimicrobial resistance genes, which can be incorporated outside species’ boundaries. The foreign DNA can come from cells (even dead ones) of the same species or from distantly related organisms. The transmitted DNA can be integrated or recombined in the recipient's chromosome or remain as an extrachromosomal inheritable element. Although we do not know much about the frequency of such *in vivo* horizontal gene transfers, genomic studies reveal that they probably account for between 5 and 43% of genes in different oral species (78). Furthermore, species of oral genera (e.g. *Actinomyces*, *Bifidobacterium*, *Fusobacterium*, *Haemophilus*, *Peptostreptococcus*, *Streptococcus*, *Porphyromonas*, *Prevotella*, and *Veillonella*) contain transposons that promote transfer of DNA between bacteria through conjugation. We also know that, for example, tetracycline use in the community not only leads to selection for the maintenance of resistance genes [*tet*(Q)] but also causes them to be transferred in the first place. This transfer is not parallel to processes that we associate with sex in higher organisms as it does not cause reproduction. Bacterial sex is simply associated with the uptake of genetic material that can be transferred vertically or horizontally to other cells. It is hardly accompanied by pleasure for the bacteria (although who knows?), unlike the ultimate goal for activities in a bordello. Therefore, dental plaque can hardly be considered a bordello in the usual context of the word, even for bacteria. However, there is no doubt that bacterial sex occurs, which here means that genes are transferred between cells. This transfer is an important mechanism for the bacteria in terms of survival in a harsh environment, for their virulence and their pathogenicity.
